# Feasibility of Non-invasive Brain Modulation for Management of Pain Related to Chemoradiotherapy in Patients with Advanced Head and Neck Cancer

**DOI:** 10.3389/fnhum.2016.00466

**Published:** 2016-09-27

**Authors:** Xiao-Su Hu, Clayton A. Fisher, Stephanie M. Munz, Rebecca L. Toback, Thiago D. Nascimento, Emily L. Bellile, Laura Rozek, Avraham Eisbruch, Francis P. Worden, Theodora E. Danciu, Alexandre F. DaSilva

**Affiliations:** ^1^Headache and Orofacial Pain Effort Lab, School of Dentistry, Department of Biologic and Materials Sciences, University of MichiganAnn Arbor, MI, USA; ^2^Center for Human Growth and Development, University of MichiganAnn Arbor, MI, USA; ^3^Division of Oral Pathology, Department of Periodontics and Oral Medicine, University of MichiganAnn Arbor, MI, USA; ^4^Department of Oral and Maxillofacial Surgery/Hospital Dentistry, University of MichiganAnn Arbor, MI, USA; ^5^Biostatistics Department, University of MichiganAnn Arbor, MI, USA; ^6^Department of Radiation Oncology, University of MichiganAnn Arbor, MI, USA; ^7^Department of Internal Medicine Oncology, University of MichiganAnn Arbor, MI, USA

**Keywords:** tDCS, EEG, head and neck cancer, pain management, chemotherapy, adjuvant

## Abstract

Patients with head and neck cancer often experience a significant decrease in their quality of life during chemoradiotherapy (CRT) due to treatment-related pain, which is frequently classified as severe. Transcranial direct current stimulation (tDCS) is a method of non-invasive brain stimulation that has been frequently used in experimental and clinical pain studies. In this pilot study, we investigated the clinical impact and central mechanisms of twenty primary motor cortex (M1) stimulation sessions with tDCS during 7 weeks of CRT for head and neck cancer. From 48 patients screened, seven met the inclusion criteria and were enrolled. Electroencephalography (EEG) data were recorded before and after tDCS stimulation as well as across the trial to monitor short and long-term impact on brain function. The compliance rate during the long trial was extremely high (98.4%), and patients mostly reported mild side effects in line with the literature (e.g., tingling). Compared to a large standard of care study from our institution, our initial results indicate that M1-tDCS stimulation has a pain relief effect during the CRT that resulted in a significant attenuation of weight reduction and dysphagia normally observed in these patients. These results translated to our patient cohort not needing feeding tubes or IV fluids. Power spectra analysis of EEG data indicated significant changes in α, β, and γ bands immediately after tDCS stimulation and, in addition, α, δ, and θ bands over the long term in the seventh stimulation week (*p* < 0.05). The independent component EEG clustering analysis showed estimated functional brain regions including precuneus and superior frontal gyrus (SFG) in the seventh week of tDCS stimulation. These areas colocalize with our previous positron emission tomography (PET) study where there was activation in the endogenous μ-opioid system during M1-tDCS. This study provides preliminary evidence demonstrating the feasibility and safety of M1-tDCS as a potential adjuvant neuromechanism-driven analgesic therapy for head and neck cancer patients receiving CRT, inducing immediate and long-term changes in the cortical activity and clinical measures, with minimal side-effects.

## Introduction

More than 50,000 Americans are diagnosed with head and neck cancer every year (Siegel et al., 2012). These patients struggle with feeding, changes in physical appearance, speech, and psychological well-being (Epstein et al., 1999; Rose-Ped et al., 2002; Sonis, 2004a). Despite advancements in treatment options, a majority of patients experience emotional and physical distress (Ichikura et al., 2016), especially during treatment as chemoradiotherapy (CRT) itself induces mucositis and excruciating local pain that impairs food intake, leading to escalating opioid overuse and, consequently, drug associated side-effects (Schaller et al., 2015). Especially, radiotherapy (RT) induces inflammation of the mouth and mucous membranes of the throat, oral-mucositis, leading to odynophagia or painful swallowing. In some cases, further dose increases do not provide analgesia (Schaller et al., 2015). These treatment-induced side-effects often result in hospitalization and breaks in treatment, which translate to lower locoregional control and survival rates (Sonis, 2004a).

Recent studies have shown the efficacy of non-invasive brain stimulation in acute and chronic pain alleviation (Hosobuchi, 1986; Nitsche and Paulus, 2000; Fregni et al., 2006; Zaghi et al., 2011; Dasilva et al., 2012; Luedtke et al., 2012; O'Connell et al., 2014; Vaseghi et al., 2014). Transcranial direct current stimulation (tDCS) is a brain stimulation technique that applies a weak direct current to the scalp that flows from anode to cathode electrodes, which tend to increase and decrease cortical excitability, respectively. Studies revealed that half of tDCS current diffuses across the scalp while sufficient current penetrates the scalp and skull to influence transmembrane neuronal potentials and modulate neuronal excitability in the cortex without eliciting action potentials (Wagner et al., 2007). The immediate effects of tDCS are due to modulation of neuronal membrane potentials at subthreshold levels, which increases or decreases the rate of action potential firing. Usually, anodal stimulation will depolarize membranes to subthreshold levels and increase cortical excitability while cathodal stimulation will hyperpolarize membranes and decrease cortical excitability (Nitsche and Paulus, 2001). Therefore, the efficacy of tDCS is influenced by parameters such as electrode position and current strength (Nitsche et al., 2005; Fregni et al., 2006). Previous studies suggest that primary cortex stimulation using tDCS was an effective tool for alleviating chronic pain. While the precise mechanism of this analgesia is unclear, growing evidence suggests that motor cortex stimulation triggers rapid phasic activation in the lateral thalamus, which results in modulation of activity in other pain related regions such as the medial thalamus, ventrolateral thalamus, insula, anterior cingulate gyrus, and upper brainstem (e.g., periaqueductal gray matter) (García-Larrea et al., 1999; Garcia-Larrea and Peyron, 2007). More specifically, lateral thalamic modulation leads to inhibition of thalamic sensory neurons, cingulate modulation leads to decreased emotional appraisal of pain, and periaqueductal gray modulation leads to descending inhibition toward the spinal cord (Garcia-Larrea and Peyron, 2007). Evidence suggests motor cortex stimulation may also cause endogenous opioid release and directly inhibit the somatosensory cortex (Garcia-Larrea and Peyron, 2007). Besides the anode placement at motor cortex, studies also suggested the prefrontal cortex (PFC) appears to mediate affective networks associated with pain (Boggio et al., 2008). Recently, our group investigated, using the μ-opioid specific radiotracer [^11^C] carfentanil, the immediate effect of conventional primary motor cortex - supraorbital (M1-SO) tDCS application in healthy subjects with positron emission tomography (PET) imaging (Mendonca et al., 2011). We demonstrated that tDCS application induced μ-opioid system activation in several pain-related regions, including the periaqueductal gray matter (PAG), dorsolateral prefrontal cortex (DLPFC) and pre-cuneus (DosSantos et al., 2012, 2014). Such findings suggest that we could potentially activate the endogenous μ-opioid system, one of the most important analgesic-related mechanisms in the brain, in head and neck cancer patients undergoing treatment, with the intent to decrease their pain suffering and improve quality of life during CRT.

Electroencephalogram (EEG) is an inexpensive and non-invasive measure of brain activity, with the advantage of high temporal resolution (milliseconds) and direct measure of neuronal activity in the human brain. EEG is frequently used to address the dynamics of brain processing of pain perception. Particular pain presents characteristically in EEG demonstration in terms of frequency and region. Moreover, using independent component analysis (ICA) and the independent component clustering (ICC) method, it is possible to estimate the stimulus evoked functional brain regions. These features greatly increase the value of using EEG in clinical pain studies. Researchers have been able to show the use of EEG in pain mechanism studies for both acute and chronic pain (Chen et al., 1983; Bromm and Lorenz, 1998; Seidel et al., 2015), including tonic cold pain (Chang et al., 2002) and chronic neuropathic pain (Bromm and Lorenz, 1998; Sarnthein et al., 2006). In addition, analgesic drugs can also trigger particular EEG alterations in the brain (Hartley et al., 2014; Graversen et al., 2015), and the pain-relieving effect varies according to individual baseline brain activity (Jensen et al., 2014).

In this feasibility study, our aim was to investigate and modulate the CRT induced mucocitis pain (inflammatory) regulatory cortical mechanisms in advanced head and neck cancer patients. While understanding central mechanisms related to pain using neuroimaging is important, it is equally important to develop novel clinical protocols aimed at relieving CRT-induced pain in head and neck cancer patients.

## Materials and methods

### Subjects

Patients with a head and neck malignancy were recruited through the University of Michigan Health System (UMHS) Department of Medical Oncology and weekly tumor board meetings. Patients were screened by the Medical Oncology clinical studies team and then approached by H.O.P.E. lab study team members for discussion of the protocol and informed consent. Inclusion criteria were (1) AJCC Stage III-IV head and neck malignancy scheduled for definitive chemoradiotherapy or radiation therapy only; (2) patients capable of understanding and adhering to the protocol requirements; (3) patients between the ages of 18–75 years. Emphasis was placed on patients with no current chronic pain conditions or use of narcotic medications; (4) all patients entered into the current protocal had biopsy confirmed head and neck squamous cell carcinoma (HNSCC). Exclusion criteria: (1) substantial dementia; (2) actively being treated for another cancer at the time of enrollment; (3) any condition that would prevent the use of tDCS, including skull abnormality, implanted metal, implanted electronic device, seizure disorder, or other neurologic conditions;(4) the use of an investigational drug or device within 30 days of study screening.

Patients were evaluated for dental clearance at the University of Michigan Hospital Dentistry Clinic as part of the pre-established standard of care. This protocol (HUM00078942) was approved by the University of Michigan Institutional Review Board. Written informed consent was obtained from all participants.

From 48 patients screened, seven met the inclusion criteria and were enrolled. Six patients were placed into the stimulation arm of the study, with the fifth patient withdrew during the third week of CRT due to CRT-related side effects (Table 1). Five patients completed the study according to the outlined protocol. The seventh patient was placed into the control arm of the study and completed the study according to the protocol, however, this patient was excluded from further EEG data analysis because of lack of sufficient data, since the signal quality in half of the data files of the specific patient failed to pass the visual data examination. A possible reason for this might be loose contact between EEG electrode and scalp. A brief report of chemotherapy agents used as well as the total radiation dose delivered can be found in Table 1.

**Table 1:** **Basic characteristics of stimulation patients**.

**ID**	**ENROLL DATE**	**AGE**	**SEX**	**GROUP**	**Disease site**	**Tobacco history**	**Quit?**	**Quit year**	**Planned cumulative radiation dose (Gy/day)**	**Carboplatin (AUC 1)**	**Paclitaxel (mg/m^2^)**	**Cisplatin (mg/m^2^)**
**1**	11/21/14	56	Male	Stimulation	Oropharynx	Yes	Yes	2014	70.00	Yes	30	N/A
**2**	12/15/14	67	Male	Stimulation	Oropharynx	Yes	Yes	1994	70.00	Yes	30	N/A
**3**	01/21/15	63	Female	Stimulation	Oral Cavity	No			66.00	No	N/A	40
**4**	02/20/15	69	Male	Stimulation	Oropharynx	No			70.00	Yes	30	N/A
**6**	03/04/15	58	Male	Stimulation	Oropharynx	Yes	No		70.00	Yes	30	N/A

For comparison we used data from two previous studies at our Institution consisting of head and neck cancer patients who did not receive tDCS or any other investigational drug or device (HUM 000221 and 000584), which was provided to us by the Department of Radiation Oncology. These two studies examined MRI techniques for patients with head and neck cancer, and only the UMHS standard of care was provided to this group, which provided additional data from a patient cohort similar to our control patient cohort. Baseline and 1-month follow-up quality of life questionnaires (UWQoL), toxicity evaluations, and weight tracking were performed for these patients, similar to baseline and 1-month follow-up information collected from patients enrolled in our study. Of the 97 patients enrolled in that study, 93 met the requirements of our study and the data from these patients were analyzed. These patients received the standard of care while undergoing CRT and served as controls for our study.

### Neuroimaging and transcranial direct current stimulation (tDCS)

**Figure 1:**
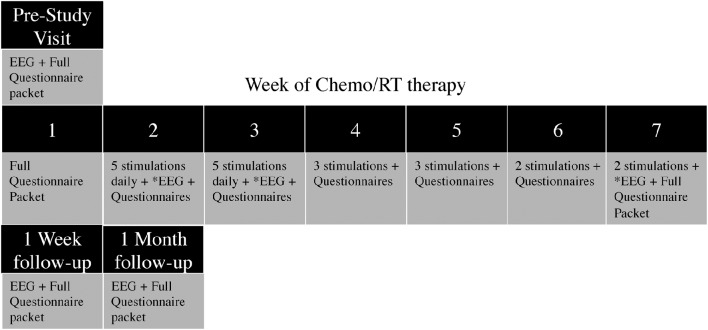
Study Protocol (^*^ denotes simultaneous stimulation and EEG recording).

Once placed into their assigned study arms, patients presented 1 week prior to the start of CRT for the pre-study visit, consisting of a 20 min EEG recording, as well as questionnaires and data collection. During the first week of CRT, only questionnaires and data collection were completed. During weeks 2 and 3 of CRT, patients received daily tDCS stimulations (five per week), and completed weekly and daily questionnaires. During weeks 4 and 5 of CRT, patients received three tDCS stimulations per week; and During weeks 6 and 7, patients received two tDCS stimulations per week. In total, 20 tDCS sessions were applied across 6 weeks (5/5/3/3/2/2)(Figure 1). EEG was recorded for 10 min prior to, during, and for 10 min after tDCS stimulation at the first appointment of the second, third and seventh stimulation weeks. Additionally, EEG recordings were taken at the 1-week and 1-month follow-up appointments. If patients were unable to complete the stimulation or missed an appointment (due to weather, emergency, holiday, etc.), the stimulation appointment was rescheduled for the day before or the day after. If two stimulations were required on the same day to make up a missed appointment, one stimulation was performed in the morning and one in the afternoon, with a minimum of 3 h in between stimulation appointments.

**Figure 2:**
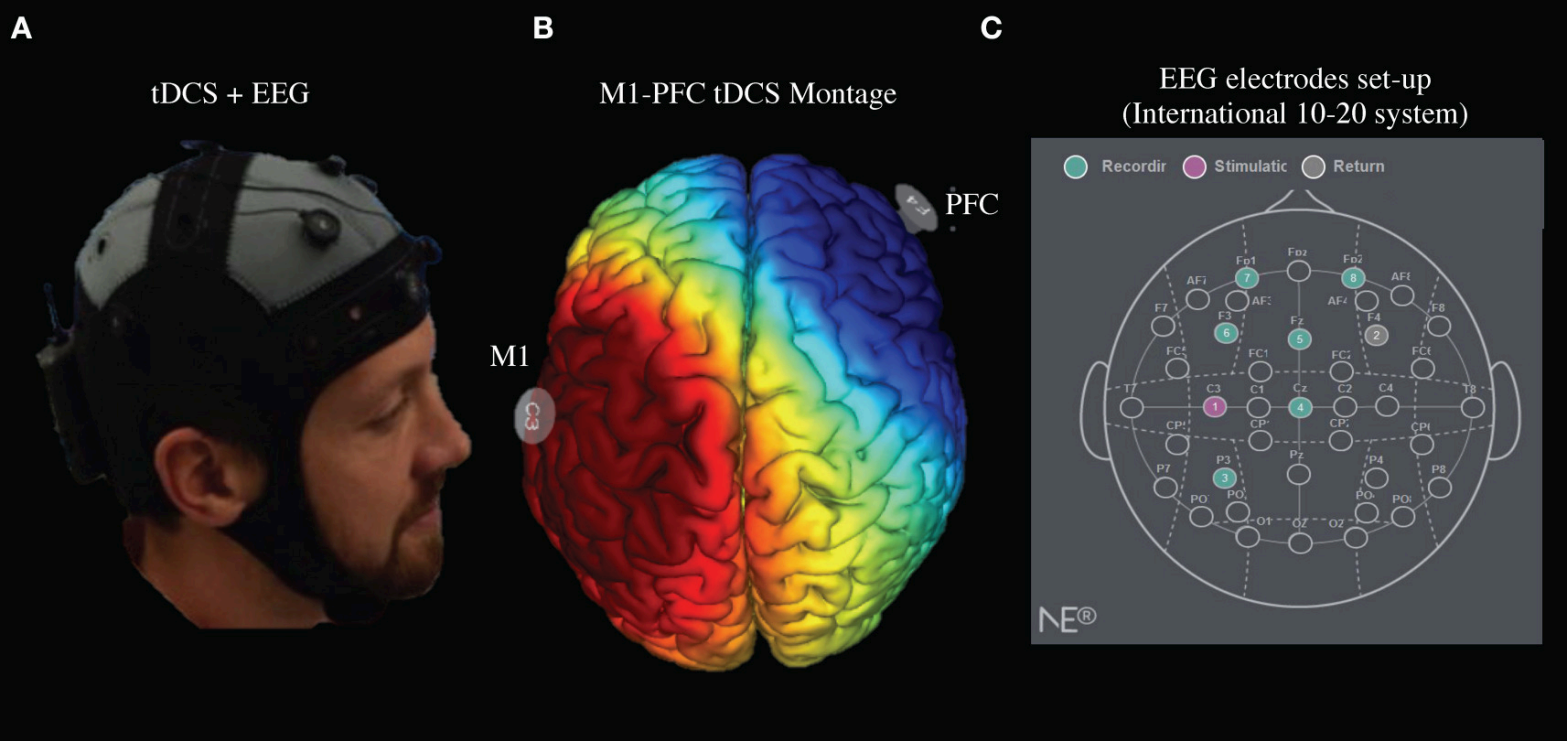
**tDCS Stimulation and EEG recording setup. (A)** M1-PFC tDCS set up with concurrent EEG. **(B)** Electric current pathway from M1 anode in red to PFC cathode in blue. **(C)** tDCS anode/cathode and EEG channel locations set up.

The 25 cm^2^ sponge-pad tDCS montage consisted of the anodal electrode placed on the left primary motor cortex (M1) at the location of C5 (according the 10–20 intermediate Modified Combinatorial Nomenclature EEG system) and the cathode electrode placed on the right DLPFC at the location of F4 (Figure 2).

Stimulation consisted of 2 mA of tDCS for 20 min, with a 30 s ramp up and cool down. During simultaneous stimulation/EEG, the stimulation electrodes were placed at C5 (anode) and F4 (cathode), and Ag/AgCl ring electrodes for EEG were placed at P3, Cz, Fz, F3, FP1, and FP2 (Figure 2). A prefabricated cap (Neuro Electrics, Spain), with previously perforated holes and a chin-strap was used to mount the electrode. The proper size cap, small, medium, or large, was determined at the pre-study visit. Approximately 6 mL of 0.9% Saline solution was used per sponge electrode for conductivity. Approximately 12 mL of Lectron II Conductivity Gel was then injected into the EEG electrode sites when applicable. Neuroelectrics StarStim (Neuro Electronics, Spain) software was used to control stimulation and EEG settings, monitor impedance and time intervals, and record EEG data.

### Pain level assessment

We selected the visual analog scale (VAS), percentage of weight loss, and common terminology criteria for adverse events (CTCAE) as indices to reflect the level of pain patients experienced during CRT. To further evaluate the tDCS treatment effect, we also used the McGill and positive and negative affect schedule (PANAS) questionnaires before and after each tDCS session. For patients in the stimulation arm, weight was measured regularly as an objective measure of nutritional status, and has been used in numerous other head and neck radiotherapy trials, including oral mucositis mitigation trials (Gellrich et al., 2015). While for patients in control arm, weight was measured at baseline and 1 month following the regular treatment. Dysphagia was recorded at weekly oncology visits for all patients in the stimulation arm. The CTCAE v3.0 has been used since 2006 at University of Michigan Health System (UMHS), and varied little from v2.0 regarding the grading of dysphagia. During the CRT process, the patients were taking oral morphine equivalent as analgesic drug.

### EEG data analysis

The EEG data analysis was completed in EEGLAB (a matlab based software, Mathworks) (Delorme and Makeig, 2004). For preprocessing, the raw data were firstly high-pass filtered at >1 Hz using basic FIR filter function. Then automatic channel rejection function was applied to reject the channel using kurtosis measure and Z-score threshold at 5. Then the filtered data were visually inspected to remove the artifacts and noisy parts. Next, the Run ICA function was used to conduct an ICA process on the data. Finally the functional EEG dipoles were estimated for each patient using DIPFIT 2.x tool in EEGLAB based on the ICs calculated from the previous step.

The post-processing steps consisted of two parts. The first part was power spectrum comparisons for all channels respectively between pre/post tDCS stimulation and between pre-study visit week/seven of tDCS stimulation. We used the Precompute channel measures and Plot channel measures functions to compare the EEG power spectra before/after as well as pre-study visit week/week 7 of tDCS. The comparison frequency bands ranged from 0 to 50 Hz. The EEG lab statistics were used and the threshold was set to be *p* < 0.05. The second part was to cluster independent components across patients and estimate the locations of group-level functional EEG dipoles. The Build pre-clustering array function was used following the Precompute component measures. The centered MNI coordinates (with a 5 mm radius voxel) of each identified IC cluster was examined for their related brain region within the automatic anatomical labeling (AAL) database (Tzourio-Mazoyer et al., 2002).

## Results

### Locations of estimated functional EEG sources

**Figure 3:**
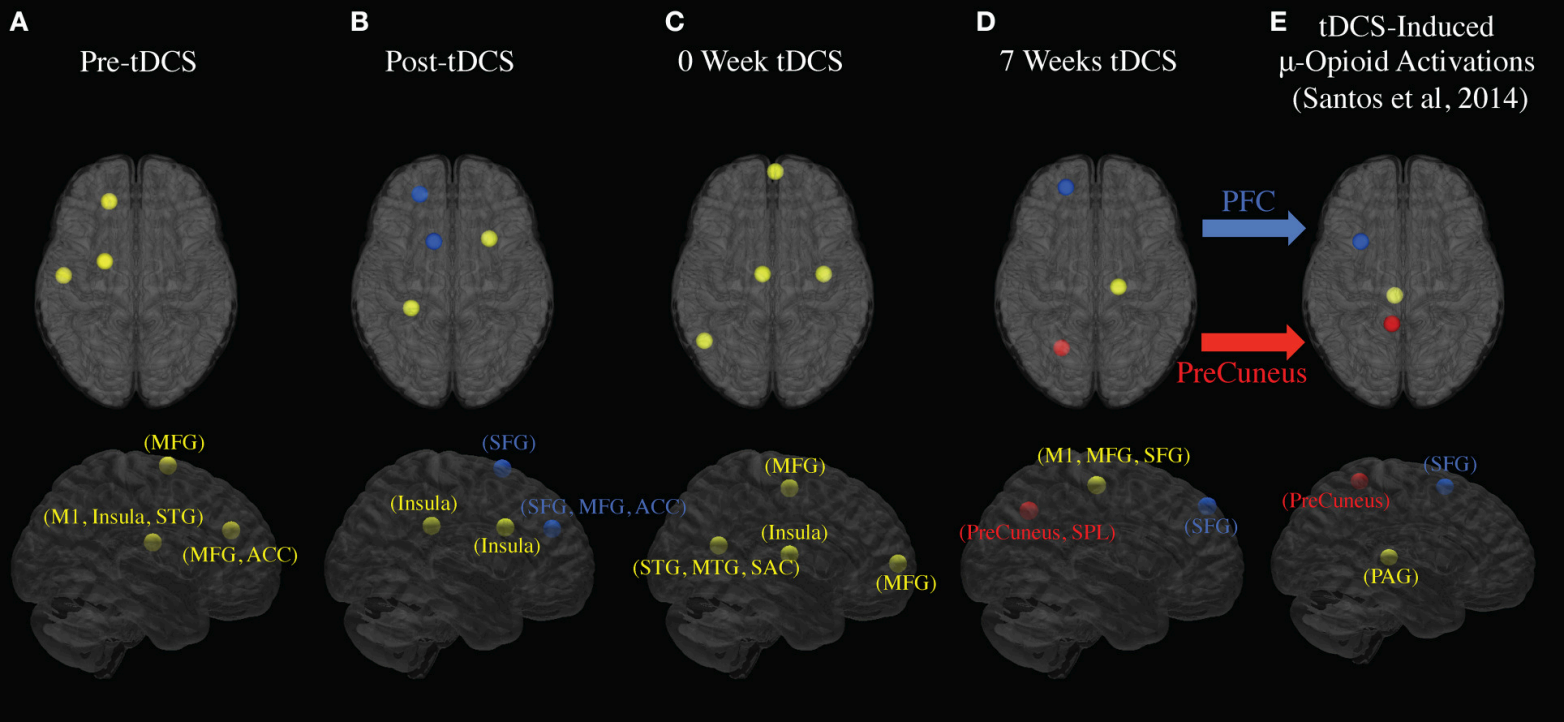
**EEG signal sources changes to PFC and PreCuneus following 7 weeks of tDCS stimulation**. The estimated locations of the EEG sources are marked out for each stage. The blue, red, and yellow dots indicate, respectively, the sources with possible locations at SFG, PreCuneus, and other areas. **(A)** Estimated signal sources before tDCS stimulation (average of week 2, 3, and 7). **(B)** Estimated signal sources immediately after tDCS stimulation (average of week 2, 3, and 7). **(C)** Estimated signal sources in pre-study visit week. **(D)** Estimated signal sources in week seven. **(E)** Estimated tDCS-induced mu-opioid activation locations (DosSantos et al., 2014).

Figure 3 shows the clustered ICs from 5 subjects by common properties of their EEG data spectrums and scalp maps. The pre-tDCS stage had three clusters separately that included estimated functional brain regions (Week 2, 3, and 7 EEG data included): left Anterior Cingulate Cortex (ACC) and left Medial Frontal Gyrus (MFG) centered at (MNI: −15, 40, 16); left MFG and Sub-Gyral centered at (−18, −2, 59); Insula, left Precentral Gyrus (primary motor cortex), and left Superior Temporal Gyrus (STG) centered at (−47, −12, 8). The post-tDCS stage had four clusters separately and included estimated functional brain regions (Week 2, 3, and 7 EEG data included): left Superior Frontal Gyrus (SFG) centered at (−9, 12, 57); left SFG, MFG, and ACC centered at (−19, 45, 17); Extra-Nuclear and Insula centered at (−47, −12, 8); Sub-Gyral, Extra-Nuclear and Insula centered at (García-Larrea et al., 1999; Zaghi et al., 2011; Hartley et al., 2014). The pre-study visit week tDCS stage had four clusters separately and included estimated functional brain regions in: Insula, Extra-Nuclear, and Sub-Gyral centered at (40, −11, 20); left STG, Supramarginal Gyrus (somatosensory association cortex), and middle Temporal Gyrus (MTG) centered at (−44, −58, 26); left MFG centered at (6, 61, 14); left MFG centered at (−3, −11, 64). The week 7 tDCS stage had three clusters separately AND included estimated functional brain regions: Sub-Gyral, right Precentral Gyrus, Middle Frontal Gyrus, and SFG centered at (20, −20, 66); left SFG centered at (−17, 50, 50); and left Precuneus and Superior Parietal Lobule centered at (−20, −63, 49). All coordinates reported were MNI coordinates.

### EEG channel spectrum analysis

**Figure 4:**
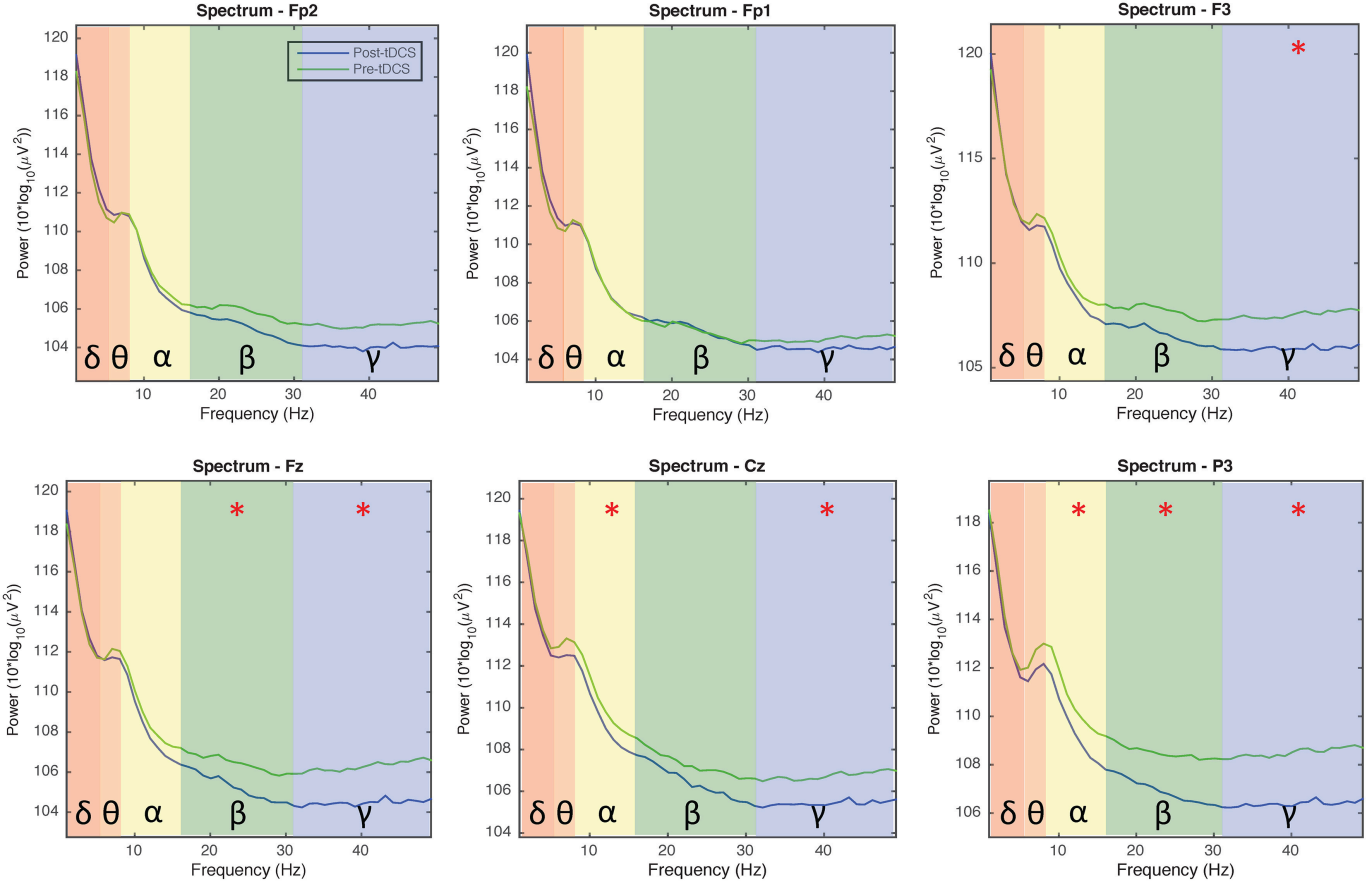
**EEG power spectra analysis results comparison for all channels before and immediately after tDCS stimulation (1 - 50 Hz; Fp1, Fp2, Fz, F3, Cz, and P3; average of week 2, 3, and 7)**. The background colors indicate EEG frequency bands: red, δ wave; orange, θ wave; yellow, α wave; green, β wave; blue, γ wave. The green and blue lines, respectively, indicate power spectra before and after tDCS stimulation. Generally the power decreased immediately after tDCS stimulation for α, β, and γ waves.

**Figure 5:**
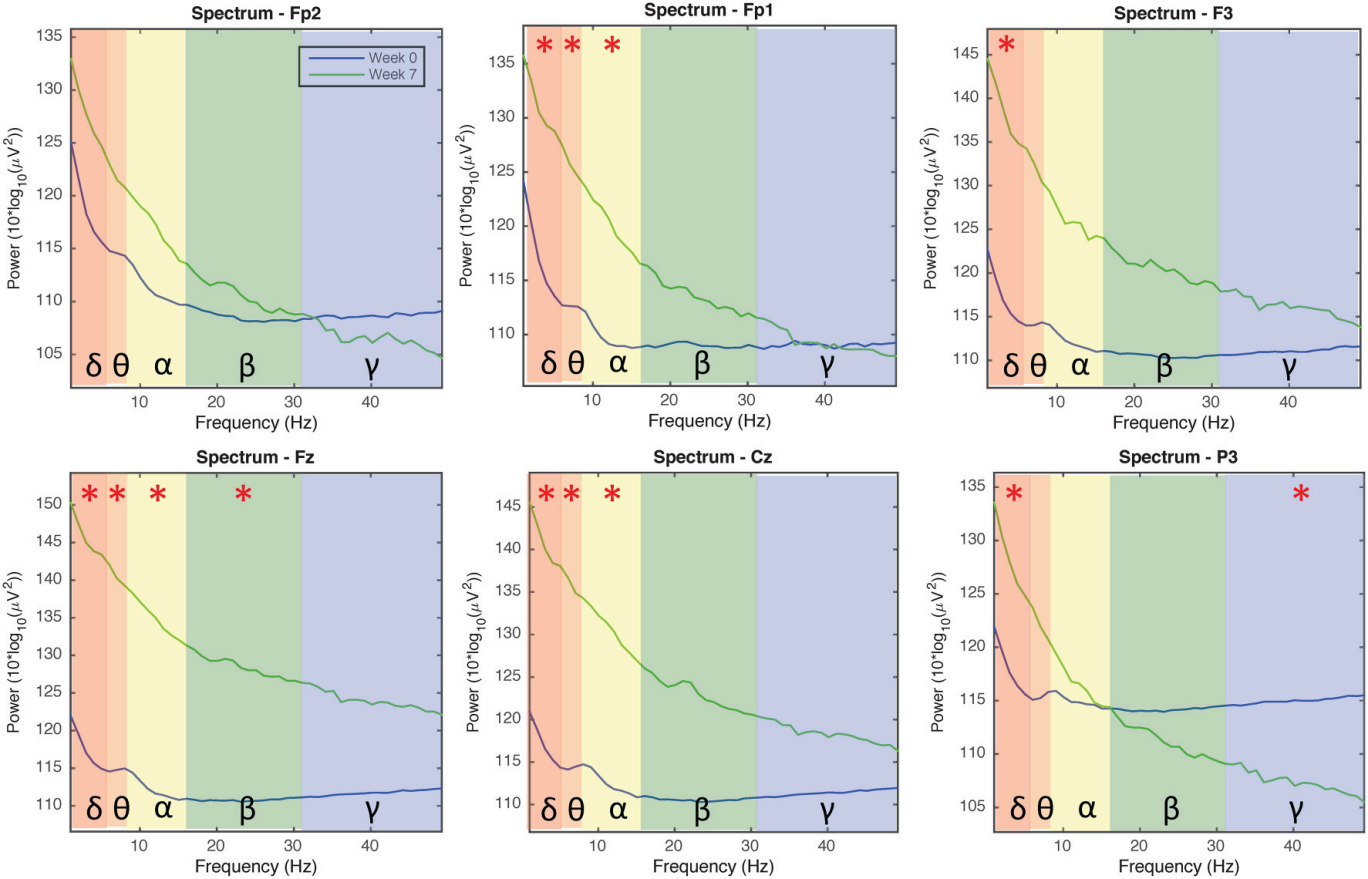
**EEG power spectra analysis results comparison for all channels in the pre-study visit week and seventh week (1–50 Hz; Fp1, Fp2, Fz, F3, Cz, and P3)**. The background colors indicate the EEG frequency bands: red, δ wave; orange, θ wave; yellow, α wave; green, β wave; blue, γ wave. The green lines and blue lines, respectively, indicate power spectra in the seventh week and pre-visit week of tDCS stimulation. Generally the power of δ, θ, α, and β waves increased, while the power of γ wave decreased in channels Fp1/Fp2/P3 and increased in channels F3/Fz/Cz, after 7 weeks of tDCS stimulation.

Figure 4 indicates EEG data spectrum change right before and after tDCS stimulus (Week 2, 3, and 7 EEG data included, power between 0 and 50 Hz frequency band were compared). Power spectra at γ band significantly decreased immediately after tDCS application at location F3, Fz, Cz, and P3 (*p* < 0.05). Power spectra at β band significantly decreased immediately after tDCS application at location Fz and P3 (*p* < 0.05). In α band, the power spectra significantly decreased after tDCS stimulus at location Cz and P3 (*p* < 0.05). Figure 5 compares EEG data spectrum in pre-study visit week and week 7 in a long term. Power spectra at δ band significantly increased after 7 weeks tDCS stimulation at location Fp1, F3, Fz, Cz, and P3 (*p* < 0.05). Power spectra at θ band significantly increased in week 7 at locations Fp1, Fz and Cz compared with pre-study visit week (*p* < 0.05). In α band, the power spectra significantly increased in week 7 at locations Fp1, Fz and Cz (*p* < 0.05). In γ band, the power spectra significantly increased in week 7 at location P3 (*p* < 0.05).

### Pain level assessment results

The pain level assessments were completed in primarily three primary measures: VAS scores, weight loss and graded dysphagia between the tDCS stimulus cohort and control cohort. Tables 2–4, respectively, show VAS scores, weight loss and graded dysphagia.

**Table 2:** **Patient-reported pain was measured using VAS at baseline (pre-visit week), week 1–week 7 of CRT process, both one-week, and 1-month follow-up**.

**ID**	**PRE**	**Wk1**	**Wk2**	**Wk3**	**Wk4**	**Wk5**	**Wk6**	**Wk7**	**WkFu**	**MoFu**
**1**	5.30	0.00	0.80	2.55	4.62	2.05	3.80	3.65	1.25	5.00
**2**	2.40	0.00	0.00	0.45	1.00	0.60	1.10	2.60	2.40	0.65
**3**	7.00	6.00	5.70	5.00	9.80	9.00	6.00	6.00	N/A	4.00
**4**	0.00	0.00	0.25	0.80	0.90	1.80	0.65	0.30	0.60	2.35
**6**	0.00	0.00	0.00	0.60	1.15	1.30	2.00	1.95	2.10	1.05
**Ave**	2.94	1.20	1.35	1.88	3.49	2.95	2.71	2.90	1.59	2.61

**Table 3:** **Patient weights along time course (Pre-visit, Week 1–Week 7, Following Week, Following month)**.

**ID**	**PRE**	**Wk1**	**Wk2**	**Wk3**	**Wk4**	**Wk5**	**Wk6**	**Wk7**	**Wk Fu**	**Mo Fu**
**STIMULATION GROUP**										
1.00	252.90	247.00	247.00	239.70	238.00	240.90	239.80	243.30	227.30	216.10
2.00	200.10	193.00	190.00	191.40	188.00	186.00	183.70	179.70	180.90	176.60
3.00	165.00	168.90	168.90	159.30	158.00	157.80	154.00	149.60	149.60	147.00
4.00	213.40	212.30	214.70	213.90	213.40	212.90	210.90	207.60	202.50	197.70
6.00	286.00	283.90	286.00	281.00	277.50	274.60	270.80	271.00	273.10	266.10
AVE (*N* = 5)	223.48	221.02	221.32	217.06	214.98	214.44	211.84	210.24	206.68	200.70
PWC%	N/A	−1.10	−0.97	−2.87	−3.80	−4.05	−5.21	−5.92	−7.52	−10.19
**CONTROL GROUP**										
AVE (*N* = 91/72)	199.95	N/A	N/A	N/A	N/A	N/A	N/A	N/A	175.58	N/A
PWC%	N/A								−12.9	

The weight variation table shows the patients' weight at each time. The weight variation in percentage table shows the variation of weight at each time based on the pre-visit week weight in percentage.

**Table 4:** **Weekly dysphagia grading of stimulation patients and control patients during CRT**.

**Group**	**Dysphagia**	**Week 1**	**Week 2**	**Week 3**	**Week 4**	**Week 5**	**Week 6**	**Week 7**
Control	0	60	34	9	4	3	0	0
	1	1	18	28	17	17	9	5
	2	0	7	21	32	33	43	39
	3	0	2	2	3	7	9	7
	4	0	0	0	0	0	0	0
	total	61	61	60	56	60	61	51
	missing	3	3	4	8	5	3	14
tDCS Stimulation	0	4	4	0	0	0	0	0
	1	0	0	3	0	0	0	0
	1/2	0	0	1	2	1	2	0
	2	0	0	0	2	2	2	3
	3	0	0	0	0	0	0	0
	4	0	0	0	0	0	0	0
	total	4	4	4	4	3	4	3
	missing	0	0	0	0	1	0	1

The Common Terminology Criteria for Adverse Events (CTCAE) version 3.0 has been used regarding to the grading of dysphagia. The selection criteria for this comparison is that the patient reported 0 score at baseline. Four stimulation patients in the intervention arm are summarized and compared to the sixty-four patients in the control arm.

The five patients who completed the tDCS protocol reported VAS at the beginning of each week. In average, 2.94 and 1.59 out of 10 were reported, respectively at baseline and 1-week follow-up.

The five patients lost 10.12, 9.60, 9.33, 5.11, and 4.51% body weight from baseline to 1-week follow-up (Table 2). One out of five (20%) patients examined in this study lost >10% of their body weight from baseline through the end of treatment. For patients in the stimulation arm, the average body weight loss was 7.52%. Of the 93 control patients examined, 72 (77.4%) lost >10% of their body weight, with a mean weight loss of 12.9%.

**Figure 6:**
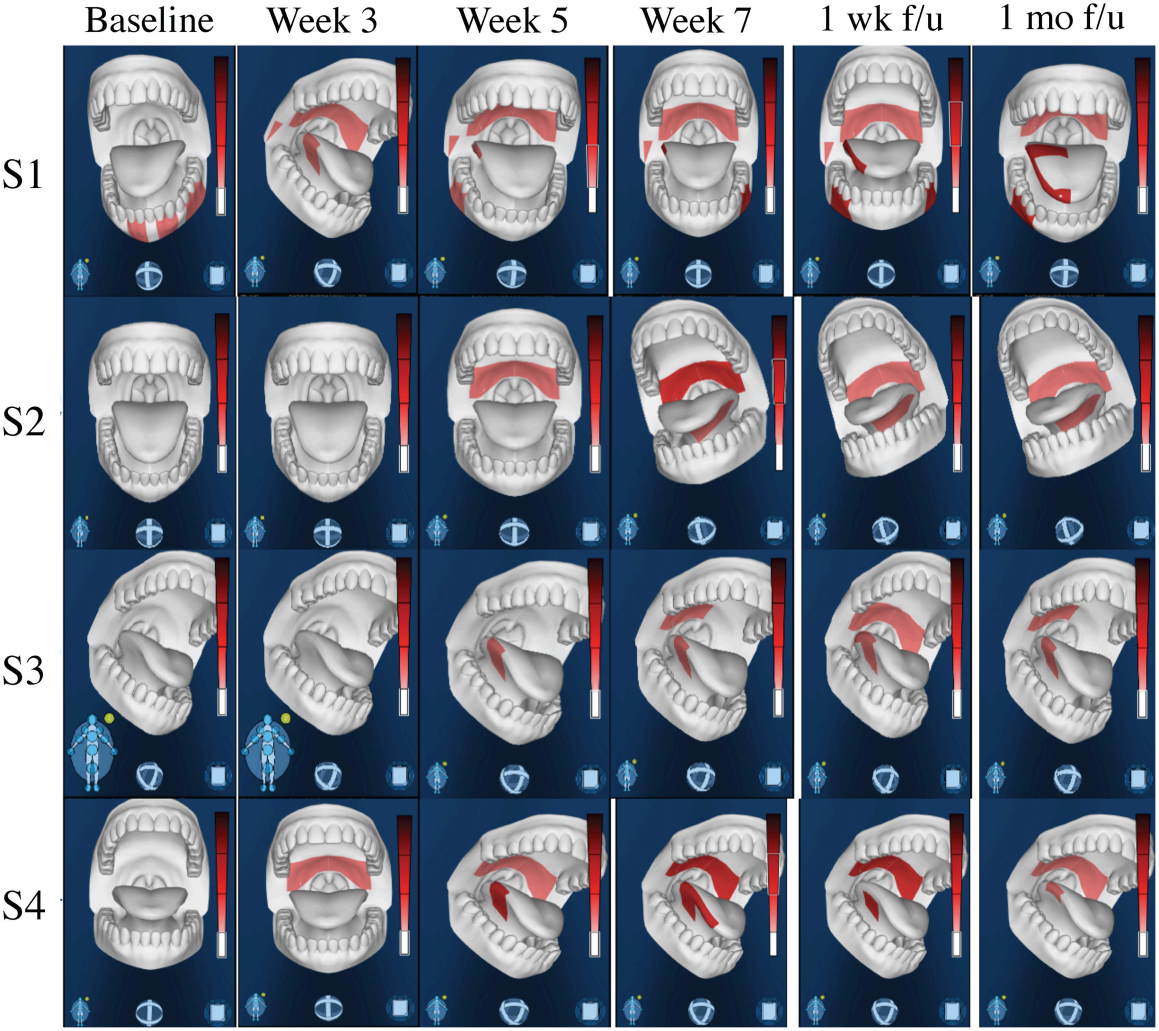
**Intraoral Pain Area and Intensity During Chemoradiation/tDCS trial**. All four patients reported only mild-to-moderate pain throughout their 7-week course of CRT. Using the GeoPain technology (MoxyTech LLC, MI), patients are able to quickly and efficiently illustrate their pain locations and pain intensity, allowing healthcare providers to both acknowledge current pain, as well as easily access and evaluate the patients pain history.

Four out of five stimulation patients reported scores of 0 at baseline, thus only control subjects with reported scores of 0 at baseline were used for optimal comparable analysis of the CTCAE grading system, and 64 of the control patients met these criteria. While none of the four stimulation patients had grade 3 dysphagia (0%), nine out of the 64 control patients that met the criteria reached grade 3 dysphagia (14.1%). Of those nine patients, some developed grade 3 dysphagia at week 2, while a majority developed grade 3 dysphagia between weeks 4 and 5, barely past their halfway mark of treatment. The difference between grade 2 (symptomatic eating/swallowing that alters eating habits) and grade 3 dysphagia (severely altered eating/swallowing habits), which lead to inadequate intake and possible indication for feeding tube placement, is clinically significant. Figure 6 shows intraoral pain area and intensity during chemoradiation/tDCS trial for all four patients receiving tDCS stimulation.

Assessment of oral mucositis grades, as assigned by the WHO Oral Mucositis Grading Criteria, revealed similar scores for both control and stimulation patients. Of the 93 control patients examined, 90 patients had mucositis grades recorded during CRT, with an average grade of 2.4 based on the worst grade given during therapy. The patients in our study had an average WHO mucositis grade of 2.5 based on the worst grade given. This suggests that tDCS has no impact on the development of mucositis in head and neck cancer patients as expected.

To further evaluate the immediate treatment effect of tDCS treatment on pain, Tables 5, 6 show the VAS, present pain intensity (PPI) and PANAS scores before and after the tDCS sessions from week 2 to week 7, respectively. Generally, the VAS reported by patients reduced in every week after the tDCS sessions (average decrease range: 0.19–0.57). While the PPI indices generally decreased one grade. Both positive and negative scores in PANAS questionnaire decreased in average after receiving tDCS stimulation (positive decrease range: −0.25–6.5, negative decrease range: 0.5–3.5).

**Table 5:** **VAS and PPI reported by patients before and after tDCS treatment in week 2–week 7**.

**ID**		**1**	**2**	**3**	**4**	**6**	**Average**
W2 BtDCS	VAS	1.68	0.00	5.88	0.10	0.06	1.54
	PPI	MI/DIC	NO	DIT/HOR	NO	NO	N/A
W2 AtDCS	VAS	0.90	0.00	5.80	0.04	0.02	1.35
	PPI	NO/MI	NO	DIC/DIT	NO	NO	N/A
W3 BtDCS	VAS	1.80	0.50	6.40	1.20	1.04	2.19
	PPI	MI/DIC	MI	DIT/HOR	NO/MI	MI/DIC	N/A
W3 AtDCS	VAS	2.14	0.44	6.22	0.58	0.76	2.03
	PPI	MI/DIC	MI	DIT/HOR	NO/MI	MI/DIC	N/A
W4 BtDCS	VAS	2.90	0.90	8.20	0.47	1.17	2.73
	PPI	MI/DIC	MI	DIT/EX	NO/MI	DIC	N/A
W4 AtDCS	VAS	1.80	0.87	8.63	0.20	1.10	2.52
	PPI	MI/DIC	MI	DIT/EX	NO	DIC	N/A
W5 BtDCS	VAS	2.27	0.67	8.90	1.07	1.00	2.78
	PPI	MI/DIC	MI	HOR/EX	MI	DIC	N/A
W5 AtDCS	VAS	0.00	0.67	9.10	0.47	0.83	2.21
	PPI	NO/MI	MI	HOR/EX	MI	DIC	N/A
W6 BtDCS	VAS	2.00	1.60	7.20	0.45	1.85	2.62
	PPI	DIC	DIC	DIT/HOR	NO/MI	DIC	N/A
W6 AtDCS	VAS	1.00	1.55	7.40	0.60	1.60	2.43
	PPI	NO/MI	DIC	DIT/HOR	NO/MI	DIC	N/A
W7 BtDCS	VAS	3.80	2.65	8.60	0.30	1.50	3.37
	PPI	MI/DIC	DIC	DIT/EX	NO	DIC	N/A
W7 AtDCS	VAS	2.20	2.45	9.05	0.35	1.15	3.04
	PPI	NO/MI	DIC	DIT/EX	NO	DIC	N/A

**Table 6:** **PANAS results reported by patients before and after tDCS treatment in week 2–week 7**.

		**ID**	**1.00**	**2.00**	**3.00**	**4.00**	**6.00**	**Average**
WEEK 2	Stim1 BtDCS	Positive	N/A	N/A	N/A	30.00	42.00	36.00
		Negative	N/A	N/A	N/A	10.00	11.00	10.50
	Stim1 AtDCS	Positive	N/A	N/A	N/A	18.00	41.00	29.50
		Negative	N/A	N/A	N/A	10.00	10.00	10.00
WEEK 3	Stim1 BtDCS	Positive	N/A	N/A	20.00	13.00	41.00	24.67
		Negative	N/A	N/A	18.00	10.00	14.00	14.00
	Stim1 AtDCS	Positive	N/A	N/A	13.00	13.00	40.00	22.00
		Negative	N/A	N/A	12.00	10.00	10.00	10.67
WEEK 4	Stim1 BtDCS	Positive	N/A	N/A	12.00	11.00	39.00	20.67
		Negative	N/A	N/A	15.00	10.00	11.00	12.00
	Stim1 AtDCS	Positive	N/A	N/A	11.00	10.00	40.00	20.33
		Negative	N/A	N/A	13.00	10.00	11.00	11.33
WEEK 5	Stim1 BtDCS	Positive	N/A	N/A	10.00	10.00	39.00	19.67
		Negative	N/A	N/A	14.00	11.00	11.00	12.00
	Stim1 AtDCS	Positive	N/A	N/A	10.00	10.00	39.00	19.67
		Negative	N/A	N/A	11.00	10.00	11.00	10.67
WEEK 6	Stim1 BtDCS	Positive	N/A	34.00	10.00	10.00	38.00	23.00
		Negative	N/A	14.00	12.00	10.00	11.00	11.75
	Stim1 AtDCS	Positive	N/A	34.00	10.00	10.00	39.00	23.25
		Negative	N/A	12.00	13.00	10.00	11.00	11.50
WEEK 7	Stim1 BtDCS	Positive	N/A	31.00	21.00	14.00	39.00	26.25
		Negative	N/A	12.00	32.00	10.00	11.00	16.25
	Stim1 AtDCS	Positive	N/A	28.00	13.00	13.00	40.00	23.50
		Negative	N/A	12.00	18.00	10.00	11.00	12.75

## Discussion

The aim of our study was to test the feasibility and safety of M1-tDCS as an adjuvant neuromechanism-driven analgesic therapy for head and neck cancer patients receiving CRT. We observed immediate superior frontal gyrus (SFG) activation in response to acute tDCS stimulation and activation of the SFG and precuneus, documented up to the seventh and final week of tDCS stimulation. In addition, power spectra analysis of EEG data showed significant changes in different frequency bands indicating possible evidence of central modulatory effect on pain. Of immediate clinical significance, the tDCS patient group showed less weight loss and dysphagia during the CRT process compared with the non-tDCS patient group, indicating less functional effects from pain for the patients in the tDCS group.

Our first finding demonstrates that long-term EEG changes induced by 7 weeks of tDCS colocalize with acute changes during tDCS stimulation observed in the endogenous μ-opioid system. In a previous study using PET, our lab used a radiotracer with specific affinity for μ-opioid receptors, [11C]carfentanil, to test the immediate pain threshold variation after applying M1-tDCS (DosSantos et al., 2012). We found that a significant increase in tDCS-induced mu-opioid receptor mediated neurotransmission in the precuneus, PAG, and left PFC. In the current study, we found the left precuneus and left PFC were activated in week 7, however, we did not observe any functional activation in PAG or other deeper regions. A possible explanation for this is that EEG as a non-invasive imaging technique produces a weaker signal from regions in the midbrain (Klein and Thorne, 2006).

We also documented a change in EEG power spectra in different frequency bands immediately and after long-term use of tDCS stimulation. Although few studies investigated clinical pain with EEG, there were several reports of EEG in different kinds of experimental pain (Chen and Rappelsberger, 1994; Ghione et al., 2005; Nir et al., 2010; Peng et al., 2015; Wang et al., 2015), however, the reported results were not consistent. α activities seem to be most commonly reported among these studies. Generally the lower amplitude of α activity indicates greater cortical excitability (Peng et al., 2015). Moreover, Wang et al. (2015) reported θ/β activity decreased in a cognitive behavior therapy group. In our study, immediately after tDCS stimulation, the α activity at Cz and P3 positions decreased significantly, indicating cortical excitability increases in the proximity of the tDCS electrodes. We also observed β activity decrease at positions Fz and P3 and γ activity decrease at positions F3, Fz, Cz, and P3. Arguably γ waves are implicated in creating the unity of conscious perception and meditation (Singer and Gray, 1995) and its sequence of heightened sense of consciousness, bliss, and intellectual acuity. Notably, meditation is known to have a number of health benefits including pain relief (Zeidan et al., 2015). In the long term, after 7 weeks of tDCS stimulation, our EEG results revealed a different pattern. α activity increased at locations Fp1, Fz and Cz, showing that cortical excitability under the path from anodal to cathodal decreased after long-term tDCS stimulation. δ/θ activities generally increased, and in channel P3 close to tDCS anodal γ activity decreased. Increased slow-wave activity, especially θ activity, and reducing fast-wave activity was detected in mental therapies for pain including neurofeedback treatment, hypnosis and meditation (Sime, 2002; Fell et al., 2010). Considering that all these therapies involved cognitive changes in the brain, it would be reasonable, based on these findings, to suggest that the increases in slower wave activity (e.g., θ and α) and decreases of faster wave activity (e.g., β) in our tDCS study, provides physical neuromodulation to reduce clinical pain. Further studies are warranted to investigate specific mechanisms of tDCS stimulation in pain relief.

Stimulation using tDCS in the current clinical study reduced patients' pain during CRT and improved quality of life. We monitored patients' weight loss and reported dysphagia as indices of pain level during their CRT. Studies have shown that during CRT for head and neck cancer, the pain level correlated with patients' weight loss and dysphagia (Gellrich et al., 2015). The average weight loss of our tDCS cohort was 7.52% compared to 12.9% of the standard of care cohort. The CTCAE graded dysphagia at grade >2 were 0% for the tDCS stimulation cohort and 14.1% for the control cohort. The severity of mucositis in our patients ranged from grade 0 to grade 4, and the amount of patient-reported pain varied greatly from patient to patient. Of the four stimulation patients examined, mucositis grades of 2 and 3 were seen, but none were scored higher than a grade 2 on the CTCAE dysphagia scale. Meanwhile, 14.1% of the 64 control patients reached scores of grade 3 on the CTCAE scale. Additionally, all four reported only mild-to-moderate pain throughout their 7-week course of CRT.

Oral Mucositis is characterized by ulceration of the mucosa, leading to pain and dysphagia, and has been reported to occur in 75–90% of patients undergoing chemo and radiation therapy for head and neck cancer (Trotti et al., 2003; Sonis, 2004a,b; Scully et al., 2006). Mucositis most commonly affects the movable mucosa, including the tongue and buccal mucosa. The pain associated with mucositis results in a significant decrease in the patients' ability to eat, swallow, and talk (Sonis, 2004a). The severity of the pain can lead to treatment breaks or dose reduction of chemotherapy (Scully et al., 2006; Elting et al., 2008). By the second week of therapy, ulcerations develop throughout the oral cavity and oropharynx, requiring opioid treatment (Trotti et al., 2003; Sonis, 2004b; Murdoch-Kinch and Zwetchkenbaum, 2011). Multiple studies and drugs are in development to relieve patients of OM, but little success has been found (Sonis, 2004b; Scully et al., 2006). Because of this, opioids are the primary method of current analgesic relief. Patients that do develop oral mucositis are four times more likely to be hospitalized due to pain and malnutrition compared to patients that do not develop OM. The symptoms can last 1–2 weeks after the end of treatment, but may last longer depending on the severity implying that head and neck cancer patients may suffer from pain and discomfort for 5–10 weeks. Although we did not see changes in oral mucositis prevalence or severity as a result of tDCS application, we noticed that tDCS application reduce pain in patients with CRT-induced oral mucositis.

Four out of the five stimulation patients analyzed had decreases in body weight < 10%, with a mean loss of 7.52% with standard deviation 2.7%, an excellent sign of long-term prognosis. The average weight loss of patients with head and neck cancer undergoing CRT was found to be 12.9% with standard deviation 5.6% in the control cohort for this study at our institution. Loss of total body weight >10% often produces higher co-morbidities and a worse prognosis in CRT patients (van Bokhorst-de van der et al., 1999; Argiris et al., 2004; Liu et al., 2006; Capuano et al., 2008). Platek et al. retrospectively reviewed 140 patients receiving chemoradio- or radiotherapy and found a median weight loss of 8.56%, classified as clinically severe (Platek et al., 2013). Ottosson et al. retrospectively examined 203 patients and found that at 5-months post-RT 77.8% of their patients suffered from weight loss >10% (Ottosson et al., 2014). Both studies emphasized multiple contributing factors to the weight loss, including dysphagia, xerostomia, radiation-induced mucositis, and other related toxicities. The mean weight loss percentages from these studies are higher than our stimulation patient average, but lower than our control average. As patients undergoing tDCS treatment may have altered pain perception, these patients may be able to better tolerate food intake, and thus report reduced weight loss at the end of therapy.

To further evaluate the tDCS treatment effect, we also used the McGill (VAS and PPI scores) and positive and negative affect schedule (PANAS) questionnaires before and after the each tDCS session. Patients generally reported reduced VAS scores and lower grade PPI indices after receiving the tDCS stimulation, indicating the tDCS has immediate effect of pain relief effect. However, the reduction scale is relatively small and due to limited number of patients, it is hard to statistically compare the scores before and after the stimulations. Both the positive and negative scores reported by patients reduced after tDCS stimulation in every week. These results indicate that cathode PFC tDCS applied in the current study was associated with analgesia for both unpleasantness and intensity ratings. Which aligned with a previous repetitive transcranial magnetic stimulation (rTMS) study that applied stimulation on DLPFC (Borckardt et al., 2011). These findings may potentially justify the DLPFC placement of tDCS stimulation.

This study has several limitations: First, the patient number recruited in the current protocol was small, leading to difficulties in statistics across patients; and second, MRI scanning information did not accompany EEG data for clustering analysis. We used a unique MNI 152 brain template for the EEG channel alignment. This may generate certain bias in group EEG functional sources location estimation. Since this was a preliminary feasibility test study, these entire limitations can and will be addressed in subsequent studies.

## Conclusion

This study gives preliminary evidence that demonstrates the feasibility and safety of M1-tDCS as an adjuvant neuromechanism-driven analgesic therapy for head and neck cancer patients receiving CRT, inducing immediate and long-term changes in the cortical activity and clinical measures, with minimal side-effects.

## Author contributions

All authors contributed to study design and manuscript writing. XH, CF, RT, TN, TD, AD contributed to data collection and analysis. AE provided information about the control cohort in the current study.

## Funding

The study was funded by MCubed (TD, LR, AD), University of Michigan.

## Disclosures

AD and Eric Maslowski are the creators of GeoPain, and also cofounders of MoxyTech LLC, which has a license agreement for the technology with University of Michigan.

### Conflict of interest statement

The authors declare that the research was conducted in the absence of any commercial or financial relationships that could be construed as a potential conflict of interest.
